# Case report: Therapeutic potential of Flourishing-Life-Of-Wish Virtual Reality Therapy on Relaxation (FLOW-VRT-Relaxation)—a novel personalized relaxation in palliative care

**DOI:** 10.3389/fdgth.2023.1228781

**Published:** 2023-08-22

**Authors:** Olive K. L. Woo, Antoinette M. Lee

**Affiliations:** Department of Psychology, The University of Hong Kong, Hong Kong, China

**Keywords:** virtual reality, palliative care, relaxation, FLOW-VRT, personalized, FLOW-VRT-Relaxation

## Abstract

In view of the global aging population and growing need of palliative care, innovative intervention for effective symptom management is of urgent need. Flourishing-Life-Of-Wish Virtual Reality Therapy (FLOW-VRT) is a brief, structured, manualized, and personalized psychological intervention with theoretical foundations based on stress coping theory, self-determination theory, flow theory, and attention restoration theory. With a specific focus on relaxation, FLOW-VRT-Relaxation intends to facilitate adaptive end-of-life coping through delivering personalized relaxation. This paper reports a case study of the application of FLOW-VRT-Relaxation, and discusses its therapeutic potential as a cost-effective method for reducing palliative symptoms by addressing patient's unmet needs. The case study is a 51-year-old Chinese female, diagnosed with advanced cervix cancer, and presented with unmet psychological (i.e., unfulfilled wishes) and physical needs (i.e., pain and fatigue) before FLOW-VRT-Relaxation. To address her unmet needs, FLOW-VRT-Relaxation was delivered by a registered clinical psychologist specialized in palliative care. Need assessment was first conducted, followed by a 10-min VR travel of Japan as her own choice. Relaxation was verbally coached during VR. Right after VR, consolidation with psychological components including psychoeducation, cognitive and emotional processing, and reminiscence intervention were delivered. The patient showed improvement in physical and psychological symptoms, lower sense of loneliness and engulfment, as well as enhanced peace after FLOW-VRT-Relaxation. The current findings provide encouraging initial support for the feasibility, acceptability, and therapeutic potential of using FLOW-VRT-Relaxation as a cost-effective, scalable and personalized VR relaxation for patients under palliative care. It is hoped that with its optimal use, FLOW-VRT-Relaxation can serve as an alternative therapeutic tool that effectively improves the end-on-life care.

## 1. Introduction

To improve the end-of-life care for patients under palliative care, a range of evidence-based interventions, both pharmacological and psychological, have been adopted (1–5). Substantial research on psychological interventions with focus on physical, psychological, spiritual, and social aspects has yielded promising results (6). Despite the ongoing practice of psychological interventions, some of the palliative needs of patients remain unmet. For instance, patients' last wishes (e.g., travelling to novel places, hiking) are often unfulfilled in view of their bedbound condition; there is inadequate private or quiet place that allows quality delivery of relaxation in inpatient setting; limited goals or pleasurable activities are available, resulting in their low sense of self-determination or control. According to a recent systematic review (7), the three most reported unmet needs of Chinese patients of advanced cancer were psychological, physical, and healthcare service and information. Another systematic review (8) more specifically identified the unmet care needs of advanced cancer patients, which included physical needs such as pain, and psychological needs such as “not being able to do the things I used to do”, “I can do less than before”, and “experiencing loss of control over one's life”. Greater unmet needs were found to be associated with “worse depression, anxiety, and quality of life, highlighting the need for interventions providing additional information and psychological support” (7).

Thanks to the advanced technology, there has been a revolution in mental health care that addresses the shortcoming and limitations of traditional treatment approach. Immersive virtual reality (VR), which is increasingly adopted in regular clinical practices, has been identified as a potentially revolutionary tool for psychological treatment of various mental disorders (9). Similar to the application of VR among mental disorders, VR shows unlimited potential in making the impossible possible in palliative care setting. Niki et al. (10) used VR technology in their pilot study to fulfil patients' dying wish of traveling, and reported significant reduction in palliative symptoms after virtual travelling, notably pain, tiredness, drowsiness, shortness of breath, depression, and anxiety. In a feasibility study, Johnson et al. (11) advocated the unlimited potential of VR as a novel treatment modality, which did not only offer quality-of-life benefits to palliative care patients, but also reduced their dependency on pharmacological treatment. Similarly, Nwosu et al. (12) echoed that VR therapy in palliative care was feasible to improve end-of-life care.

Underlying explanations for VR's treatment effect among palliative care patients have been preliminarily explored. The first possible reason relates to the concepts of distraction. A pilot study conducted by Lloyd and Haraldsdottir (13) reported on participants' positive experience of VR intervention among palliative care patients, including reports of joy and happiness and “being lifted out of their current situation” (p. 348), and therefore VR sessions “were able to offer people the capacity for respite from emotional suffering as participants found their experiences to be joyful and calming ” (p. 349). Other studies also reported consistent feedback from participants on the sense of being away from their current situation, which may have relieved their physical and psychological symptoms (13–15). A sense of escapism during immersive VR is also proposed, which leads to reduced palliative symptoms, including pain, anxiety, and fatigue (10, 11, 16–20). In terms of pain distraction, VR has been appraised as an alternative form of analgesia, attributed to the successful management of pain and emotional distress and anxiety symptoms among patients (10, 11, 16–19, 21–25). As relaxation techniques were reported to be the second most frequently used of all complementary and alternative medicine modalities by cancer patients receiving treatment (26), VR with natural scenes were found to provide relaxation both objectively and subjectively (27).

Despite the preliminary exploration of VR studies among palliative care patients, there remains a significant research and clinical gap. Nwosu et al. (12) reported that no VR resources have been validated for the specific purpose of symptom relief in palliative care, and it is crucial to “consider the purpose of the activity, to identify how content is developed, and to define how (and by whom) it is delivered” (p. 3). Ma et al. (28) emphasized the need of standardization of programs and procedures to facilitate the routine use of VR-based psychotherapies. Regarding relaxation as common practice for symptom control, Pizzoli et al. (29) advocated that personalized approach of VR with focus on personalized needs brings economic advantages and effective interventions for specific patients; however, personalized VR could be difficult to implement within organizational practices, such as hospital. In addition, VR interventions encountered a common problem that compromises its effectiveness and wide adoption- high production cost and time for personalized content. Coelho et al. (30) described the person-centered approach of VR in the context of reminiscence as “more strenuous, as it requires interviewing, traveling, and filming, taking into account the participants’ profile. This may limit the possibility of conducting similar interventions at a larger scale. Nevertheless, a more person-centered approach, which recognizes the individual's biography, is more likely to promote engagement and well-being” (p. 10). Although the clinical benefits of personalized VR have been well acknowledged (31), there is not a single VR study targeting the personalized and scalable use of VR relaxation in palliative care setting.

To address both the research and clinical gap, a novel virtual reality psychological intervention, which is referred as *Flourishing-Life-Of-Wish Virtual Reality Therapy* (FLOW-VRT®) specially designed for patients under palliative care was developed in the current study. FLOW-VRT is a brief, structured, manualized, and personalized psychological intervention with theoretical foundations based on flow theory (3), stress coping theory (1), self-determination theory (2), and attention restoration theory (4, 5). FLOW-VRT intends to relieve physical and emotional distress, and to promote quality-of-life. It is a standardized and manualized psychological intervention with thorough consideration on the purpose of each VR experience, which VR content to be selected, how VR is to be delivered, and by whom it is delivered. FLOW-VRT with focus on relaxation (FLOW-VRT-Relaxation) allows patients to choose their VR relaxation content, while addressing their unmet physical and psychological needs. A randomized controlled study is currently underway to compare the effectiveness of FLOW-VRT-Relaxation with traditional relaxation practice in palliative symptom control. The current study aims to develop, and test the feasibility and usability of FLOW-VRT-Relaxation as a new protocol, for its potentials to personalized yet be scalable and cost-effective. The current case study documents the treatment response of the first patient under palliative care who receives FLOW-VRT-Relaxation in a hospital setting. The patient is a 51-year-old Chinese female diagnosed with cervix cancer, admitted to hospital due to worsening functional status with unresolved pain and unfulfilled wish.

## 2. Materials and methods

### 2.1. Participant

Ms. W, 51-year-old Chinese female was diagnosed with cervix cancer and depression in 2012. She was later diagnosed with right sciatic nerve sheath tumor in December 2022. She was admitted to a palliative inpatient ward of a public hospital due to worsening functional status with fungating tumor in September 2022. Please refer to Supplementary Table S1 for the timeline showing episode of care. During admission, she received medication and chemoradiotherapy. Prior to admission, she was unemployed, and mostly homebound relying on rollator for indoor activities and wheelchair for outdoor activities. She was educated to tertiary level.

Upon approach, she was oriented and alert with stable mood, congruent affect, coherent and relevant speech. Despite medical prescriptions (i.e., Etoricoxib, Pregabalin, Morphine and Methadone), she complained about pain and tiredness still. She showed acceptance to current palliative status, aiming for minimal physical and emotional suffering during end-of-life stage. She expressed her long-standing wish of travelling to Japan where she had not visited before, and likely would not be possible to visit in the future. The main objective of the palliative care team was to minimize her suffering looking specifically at her physical, psychological, social and spiritual challenges. She had never used VR technology prior to the study.

### 2.2. Therapist

The first author of this paper (Woo) delivered FLOW-VRT-Relaxation to Ms. W. Woo is a registered clinical psychologist under Hong Kong Institute of Clinical Psychologist, and certified thanatologist under Association of Death Education and Counselling. She was also a candidate of the Doctor of Psychology (Clinical Psychology) in the University of Hong Kong. She was chiefly responsible for the delivery of psychological services in a palliative care unit of a rehabilitation hospital.

### 2.3. Design and study procedure

The current case study used a within-subject design. Need assessment was first conducted using a need assessment schedule. The schedule was developed in current study to optimize the use of VR by assessing patient's needs for relaxation. A list of most popular VR relaxation content, which were the results of survey^1^ conducted among patients under palliative care, was shown to Ms. W who was then asked to choose her preferred relaxation content. She chose Japan Sakura as her virtual relaxation content. A Youtube video on Sakura was retrieved for her virtual travel (https://www.youtube.com/watch?v=Awz-wNJ_bk0&list=PLztGxkyfoE6_TbFMscEDAbfdDEdV2InD3&index=9&t=13s). Meta Quest 2 as the head-mounted display was used for her virtual exposure.

In order to facilitate the exploration of the virtual environment, a semi-structured protocol consisting of verbal cues and questions was followed during VR experience. This protocol included verbal instructions and questions such as: “Please relax and look around you”; “What are you looking at?”; and “How do you feel now?”. Ms. W was allowed to express freely her thoughts and emotions with reassurance at first, after which she was verbally coached on relaxation exercise. The VR lasted for 10 min.

After VR, consolidation with psychological components was delivered. It aimed to enhance self-esteem, facilitate adaptive coping, and build resilience. It included (1) psychoeducation, i.e., elaborating on the relationship between physiological response and mood; (2) cognitive and emotional processing on the virtual experience; and (3) reminiscence intervention that facilitated sharing on her recollections associated with the VR travel.

The baseline assessment was taken right before the need assessment, while the post-intervention assessment was conducted immediately after the consolidation. The whole session was delivered by the registered clinical psychologist, who is also the principal investigator of the current study.

### 2.4. Assessment

*Chinese version of the Edmonton Symptom Assessment System* (CESAS) (32, 33) was used in the current study as a measure of nine common symptoms experienced by cancer patients: pain, tiredness, nausea, depression, anxiety, drowsiness, appetite, well-being and shortness of breath. It has been shown to be reliable for symptom assessment in patients undergoing palliative care (34).

*UCLA Loneliness Short* (35), as an abbreviated version of the *20-item Revised UCLA Loneliness Scale* (36), is a 3-items scale used to rate the degree of loneliness by asking how often patients feel they lack companionship, feel left out, and feel isolated from others. Each item is rated from 0 to 3 on a numerical scale with 0 indicating *Not at all*, and 3 indicating *Very often*. Its Cantonese version (37) was used in this study.

Illness Identity Questionnaire (38) is a validated questionnaire to measure four aspects of illness identity: engulfment, rejection, acceptance, and enrichment. To address the research question, only the 8-item engulfment subscale was used. Each item is rated from 1 to 5 on a numerical scale with 1 indicating Strongly Disagree, and 5 indicating Strongly Agree. Standard translation-back translation procedure was used to translate it into Cantonese Chinese.

*Functional Assessment of Chronic Illness Therapy- Spiritual Wellbeing Scale* (39) is a validated questionnaire to measure aspects of spirituality and/or faith that contributes to improving quality of life. It consists of 12 items which fall under three domains: meaning, peace, and faith (40–42). Each item is rated from 0 to 4 on a numerical scale, with 0 indicating *Not at all*, and 4 indicating *Very much*. Only the 4-item peace subscale was used in this study to address the research question (i.e., whether VR exposure can enhance sense of peace), and to avoid overloading patients by keeping a realistic questionnaire length. Its Chinese version (43) (retrieved from https://www.facit.org/measures/FACIT-Sp-12) with granted license was used in this study.

Demographic and clinical variables, e.g., age, sex, education, medical diagnosis and treatment, were collected by retrieving from the medical records as well.

## 3. Results

Ms. W's self-rating of outcome measures are shown in Supplementary Table S2. FLOW-VRT-Relaxation was found to improve her palliative symptoms, i.e., reduced physical and emotional symptoms, sense of loneliness and engulfment, and increased level of peace. During consolidation phase, i.e., psychological interventions delivered after VR exposure, Ms. W reported that since she hadn't visited Japan before, such VR experience gave her the special opportunity to take a look at Japan. Surprisingly, it did not look like Japan that she imagined, but like Cheung Sha Wan, i.e., a city place in Hong Kong. Japan turned out to be less beautiful than she thought, which she found interesting to know. She was aware that she was physically in Hong Kong, but she “felt” like being there in Japan during VR exposure. Such VR exposure reminded her of her beloved home village in rural area of China where she had her most favorite food and trees. She would recommend VR to others as it carried no negative effects but positive experiences to her.

## 4. Discussions

In face of the mounting need of palliative care across the globe, timely intervention for effective symptom management is of pressing need. Despite the emergent clinical and research evidence for the efficacy and usability of VR relaxation in palliative care, there remains a big challenge of producing personalized, scalable and cost-effective VR for its wide adoption.

To address the clinical need, the current study developed a manualized and personalized VR relaxation intervention named FLOW-VRT-Relaxation as a novel psychological intervention in palliative care. It intends to fulfill patients' last wishes, facilitate sense of control, and allow immersive relaxation experience. Capitalized on the freely-available VR video, the current case study reveals the unlimited potential of delivering personalized FLOW-VRT-Relaxation with zero production cost and minimal time. Novel elements of FLOW-VRT-Relaxation include need assessment before VR, personalized VR relaxation content, real-time relaxation coached by a registered clinical psychologist, and psychological interventions delivered right after VR that help to consolidate the VR experience and enhance therapeutic effects.

Patient's responses in the current study appeared to be positive. After FLOW-VRT-Relaxation, the patient reported reduced physical and emotional symptoms, sense of loneliness and engulfment, as well as increased level of peace. Patient particularly highlighted the uniqueness of such intervention that provided a special opportunity to virtually fulfill her wish by “feeling” like travelling to Japan, with realization that her hometown was not less beautiful. Such a personalized VR experience appeared to address not only her unmet physical needs (e.g., pain), but also her psychological need of wish fulfillment in her end-of-life stage.

The current study has several limitations. Since it is impossible to make broad scientific conclusions based on the results of a single patient (44), case studies must be followed up with controlled studies. Although there are limitations in reporting findings from one single case, the positive experience of the patient aligned with those from previous research into VR traveling among patients under palliative care (13–15). The results of the current feasibility study indicate that FLOW-VRT-Relaxation has potential without observation of contraindications. In light of the encouraging outcomes, in the next phase of this research program, we will begin to assess clinical outcome with a randomized controlled trial and a control arm.

Another limitation is that the patient was receiving medical treatment that might have led to improved symptoms. The use of a within-subjects design measuring outcomes right before and after FLOW-VRT-Relaxation was designed to isolate the effects of FLOW-VRT-Relaxation. Furthermore, since the death of patients under palliative care can be imminent, i.e., within a few hours or days (45), the present study only measured the short-term effect of FLOW-VRT-Relaxation. Although this patient reported immediate short-term benefits, long-term effect or skill development would be worthy of further investigations, e.g., continual relaxation practice during rehabilitation journey even without access to virtual reality.

Despite limitations, the current findings provide encouraging initial support for the feasibility, acceptability, and clinical potential of using FLOW-VRT-Relaxation under palliative care. With appropriate trainings, future implementation leaders may help increase the accessibility of such cost-effective psychological interventions in wider populations across different settings. It is hoped that with its optimal use, FLOW-VRT-Relaxation can serve as an alternative therapeutic tool that effectively improves the end-on-life care by addressing patients' physical and psychological needs.

## Data Availability

The raw data supporting the conclusions of this article will be made available by the authors, without undue reservation.

## Footnotes

^1^A prior survey was conducted interviewing patients under palliative care to ask them to report their most preferable places for relaxation. The results were collected till data saturation. The most popular relaxation places were scenery of mountain, lake, beach, forest, Japan Onsen, Japan Sakura, sky, and underwater world. Patients were also given the option “others” if they have preferable relaxation places other than the aforementioned eight places.
