# Supplementary material for: Case report: Therapeutic potential of Flourishing-Life-Of-Wish Virtual Reality Therapy on Relaxation (FLOW-VRT-Relaxation)—a novel personalized relaxation in palliative care
Source: Front Digit Health. 2023 Aug 22;5:1228781. doi: 10.3389/fdgth.2023.1228781 (PMC10477913; doi:10.3389/fdgth.2023.1228781)
Supplement: Supplementary file 1 [file Table1.docx]

Supplementary Material

Case report: therapeutic potential of

Flourishing-Life-Of-Wish Virtual Reality Therapy on Relaxation (FLOW-VRT-Relaxation)

—a novel personalized relaxation in palliative care

Olive K. L. Woo*, Antoinette M. Lee.

*** Correspondence:** Olive K. L. Woo: [wkl049@ha.org.hk/](mailto:wkl049@ha.org.hk/) [olivewookitling@gmail.com](mailto:olivewookitling@gmail.com)

# Figures and Tables

## Table 1

Timeline showing episode of care

| **Episode of Care** | **Date** |
| --- | --- |
| Admission to palliative care unit | Late September 2022 |
| Prescribed medications | Late September 2022 (i.e., since admission) |
| FLOW-VRT-Relaxation | Late December 2022 |

**1.2 Table 2**

Participant’s self-rating of outcome measures

|  |  | **Score Range** | **Pre-score** | **Post-score** |
| --- | --- | --- | --- | --- |
| 1. **CESAS** | | | | |
|  | Pain | 0-10 | 4 | 0 |
|  | Tiredness | 0-10 | 5 | 0 |
|  | Nausea | 0-10 | 0 | 0 |
|  | Depression | 0-10 | 2 | 0 |
|  | Anxiety | 0-10 | 0 | 0 |
|  | Drowsiness | 0-10 | 6 | 0 |
|  | Loss of Appetite | 0-10 | 4 | 0 |
|  | Well-being | 0-10 | 0 | 0 |
|  | Itching | 0-10 | 0 | 0 |
|  | Shortness of breath | 0-10 | 0 | 0 |
|  | Physical Subscale | 0-60 | 19 | 0 |
|  | Emotional Subscale | 0-20 | 2 | 0 |
|  | Total | 0-100 | 21 | 0 |
| 1. **Loneliness** | | | | |
|  | Total | 0-3 | 3 | 0 |
| 1. **Peace** | | | | |
|  | Total | 0-16 | 10 | 16 |
| 1. **Engulfment** | | | | |
|  | Total | 0-40 | 40 | 16 |
